# Molecular Hybridization of Naphthoquinones and Thiazoles: A Promising Strategy for Anticancer Drug Discovery

**DOI:** 10.3390/ph18121887

**Published:** 2025-12-13

**Authors:** Leonardo Gomes Cavalieri de Moraes, Thaís Barreto Santos, David Rodrigues da Rocha

**Affiliations:** Instituto de Química, Universidade Federal Fluminense, Outeiro de São João Batista s/n°, Niterói CEP 24020-141, RJ, Brazil; leonardocavalieri@id.uff.br (L.G.C.d.M.); thaisbarreto@id.uff.br (T.B.S.)

**Keywords:** cancer, naphthoquinones, thiazole, molecular hybridization, anticancer activity

## Abstract

Cancer remains one of the leading causes of morbidity and mortality worldwide, demanding the continuous search for novel and more selective chemotherapeutic agents. Quinones, particularly naphthoquinones, constitute a privileged class of redox-active compounds with well-documented antitumor activity. Likewise, thiazoles represent a heterocyclic scaffold widely explored in medicinal chemistry due to their broad pharmacophoric adaptability and diverse biological activities. In this context, this review comprehensively explores the chemical synthesis and anticancer potential of hybrid molecules combining the naphthoquinone and thiazole scaffolds. The hybridization of these pharmacophores has emerged as a powerful strategy to design multitarget antitumor agents. The review summarizes key synthetic methodologies, including Hantzsch, hetero Diels–Alder cycloaddition and multicomponent reactions, leading to structurally diverse hybrids. Particular emphasis is placed on derivatives exhibiting strong cytotoxic effects against a broad spectrum of cancer cell lines (e.g., OVCAR3, MCF-7, A549, HCT-116, HeLa, and Jurkat), low toxicity toward normal cells and well-defined mechanisms of action involving topoisomerase IIα, EGFR, STAT3, and CDK1 inhibition, as well as ROS generation and cell cycle arrest. Among these, certain hybrids displayed nanomolar potency and high selectivity indices, reinforcing their potential as promising lead compounds for anticancer drug development.

## 1. Introduction

Cancer comprises more than one hundred complex and heterogeneous diseases arising from the accumulation of genetic mutations that disrupt cellular growth control [1,2,3]. Mutation is the central event in carcinogenesis, affecting genes regulating cell growth, DNA repair machinery, and genes controlling apoptosis [4,5]. Cancer is one of the main representatives of the group of noncommunicable diseases [6], responsible for 19.3 million new cases and 10 million deaths in 2020, and the main public health problem in the world [7,8]. Despite great geographic variability, breast cancer is the most common type of cancer among women in approximately 80% of countries. In men, there is considerable international variability, with lung, prostate, and liver cancers being the most common [9].

Chemotherapy is one of the most commonly used methods and is often performed in combination with other types of treatment [10,11]. However, the toxicity of drugs used in chemotherapy in healthy tissues limits the dose that can be administered, just as pharmacokinetic factors limit the amount that can reach the tumor [11,12]. The most important limitation to the success of chemotherapy is tumor resistance to drugs [13,14]. This resistance may be intrinsic or acquired and can arise from several mechanisms, including increased drug efflux, altered drug metabolism, enhanced DNA repair, and target mutations. One of the most common resistance mechanisms is the expression of efflux pumps, such as P-glycoprotein, reducing intracellular drug [15,16,17]. Due to the heterogeneous nature of the tumor, the use of more than one drug, with different pharmacodynamic characteristics, for treatment becomes common [14,18].

Cancer is seen as a disease capable of causing a major impact on society due to its mortality and morbidity rates. Thus, the search for new anticancer molecules remains an urgent priority. Hybrid molecule strategies have emerged as a promising approach to rational drug design, whether in the search for substances with more potent or more selective activity [19,20]. These molecules can be obtained by mixing pharmacophoric groups or even by combining entire drugs [21]. The presence of two moieties with different biological targets provides the possibility of combining drugs into a molecule that exhibits greater efficacy, lower toxicity, and less vulnerability to the development of cancer resistance [22].

**Figure 1:**
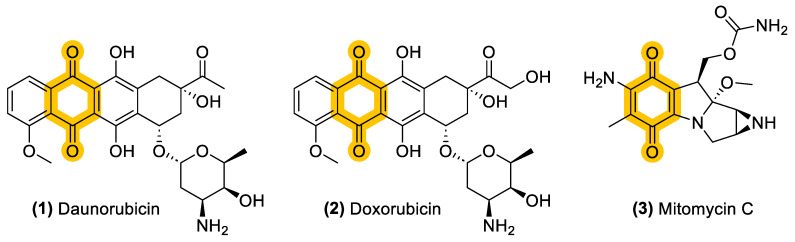
Examples of antitumor drugs containing quinonoid moiety.

Within this context, quinones are widely recognized in the literature for their diverse biological activities, including antineoplastic [23,24,25,26,27,28], antiviral [29,30,31], antifungal [32,33,34], antibacterial [35,36,37], and antiparasitic [38,39,40] effects. Clinically relevant quinonoid drugs include the anthracyclines, such as daunorubicin (**1**) and doxorubicin (**2**) (Figure 1), one of the most widely used antitumor drugs in the world, as well as mitomycin C (**3**) [41].

Quinones can exert their biological activity through different mechanisms, which is a consequence of the structural diversity present in this family of substances. For example, the mechanisms of topoisomerase inhibition [42], inhibition of indoleamine-2,3-dioxygenase [43] inhibition of the P2X7R ion channel [27], DNA alkylation and inhibition of heat shock proteins have already been studied [44,45]. A mechanism of action that tends to be common to molecules in this family is the generation of reactive oxygen species (ROS) within the target cell, causing damage to DNA and other macromolecules, which culminates in apoptosis [46].

**Scheme 1:**
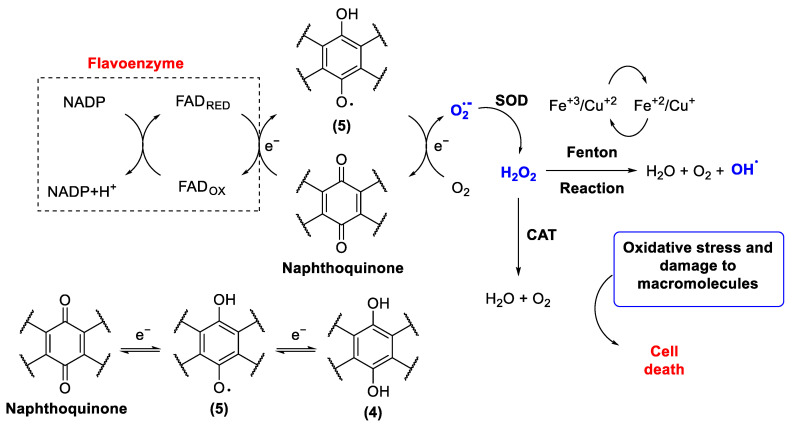
Summary of the redox cycle for ROS generation.

Quinones can undergo two-electron reduction to form hydroquinones (**4**), or one-electron reduction to form semiquinone radicals (**5**) through reductases. After this reduction, a cyclic process occurs in the presence of oxygen, which mainly produces superoxide radicals (O_2_^•−^). The superoxide radical is then converted to peroxide by the superoxide dismutase (SOD) enzyme. At this stage, the peroxide can be transformed into water and singlet oxygen by the catalase (CAT) enzyme, or it may undergo the Fenton reaction, forming hydroxyl radical (•OH). The ROS generated in this cycle creates oxidative stress in the cell, causing the scission of DNA molecules, unsaturated lipids, and proteins, and, consequently, leading to cell death (Scheme 1) [47].

**Figure 2:**
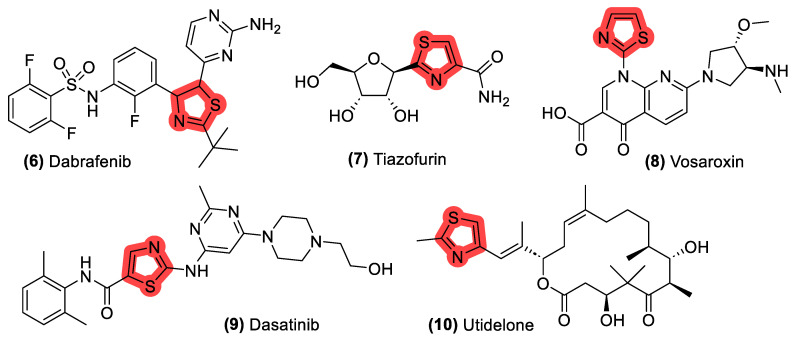
Examples of antitumor drugs containing the thiazole moiety.

Similarly, the thiazole scaffold is a crucial building block used in medicinal chemistry for the synthesis and preparation of new compounds with a variety of biological activities, such as antidiabetic [48,49], antihypertensive [50,51], analgesic [52,53], neuroprotective [54,55,56], antiviral [57,58], antiparasitic [59,60], antifungal [61,62], antimicrobial [63,64], and antiproliferative [65,66,67,68]. The thiazole moiety is present in a wide variety of pharmacological classes of antitumor drugs (Figure 2), such as dabrafenib (**6**), an inhibitor of enzyme B-RAF; tiazofurin (**7**), a dehydrogenase inhibitor; vosaroxin (**8**), a topoisomerase inhibition; dasatinib (**9**), a Bcr-Abl tyrosine kinase inhibitor; and utidelone (**10**), a microtubule inhibitor [69]. Due to its privileged structure, the thiazole ring can interact with multiple molecular targets relevant to cancer therapy [70], such as EGFR kinase inhibition [71], Akt (PKB) protein kinases inhibition [72], topoisomerase inhibition [73], and inhibition of tubulin polymerization [74].

Given the strong anticancer potential of both quinone and thiazole pharmacophores, combining these two privileged structures into hybrid molecules represents a logical and promising strategy for developing new chemical entities with enhanced biological activity.

## 2. Quinones–Thiazole Hybrids and Anticancer Activity

**Scheme 2:**
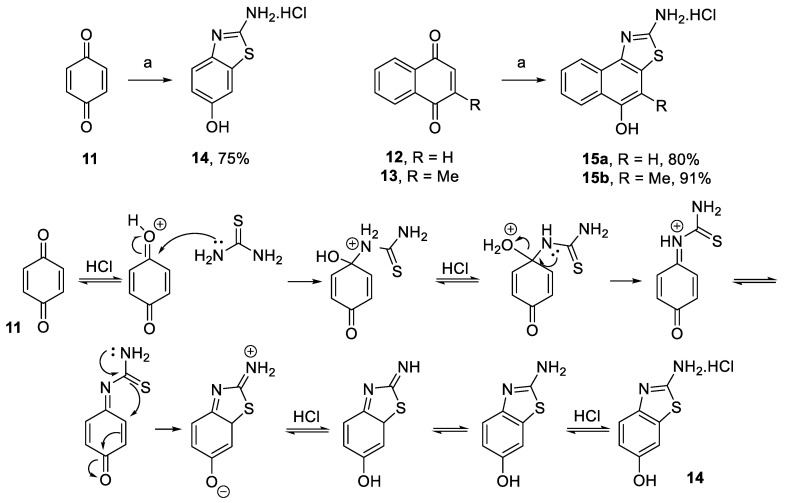
First synthetic hybrids of quinones and thiazoles: (a) thiourea, EtOH, conc. HCl, rt.

The first experiments for the synthesis of hybrid molecules of quinones and thiazoles were reported in the early 1970s, involving the between thiourea and excess 1,4-benzoquinone (**11**) or 1,4-naphthoquinones (**12**,**13**) to produce 2-amino-6-hydroxybenzothiazoles (**14**) and 2-amino-5-hydroxynaphtho[1,2-*d*]thiazoles (**15**), respectively (Scheme 2) [75].

**Scheme 3:**
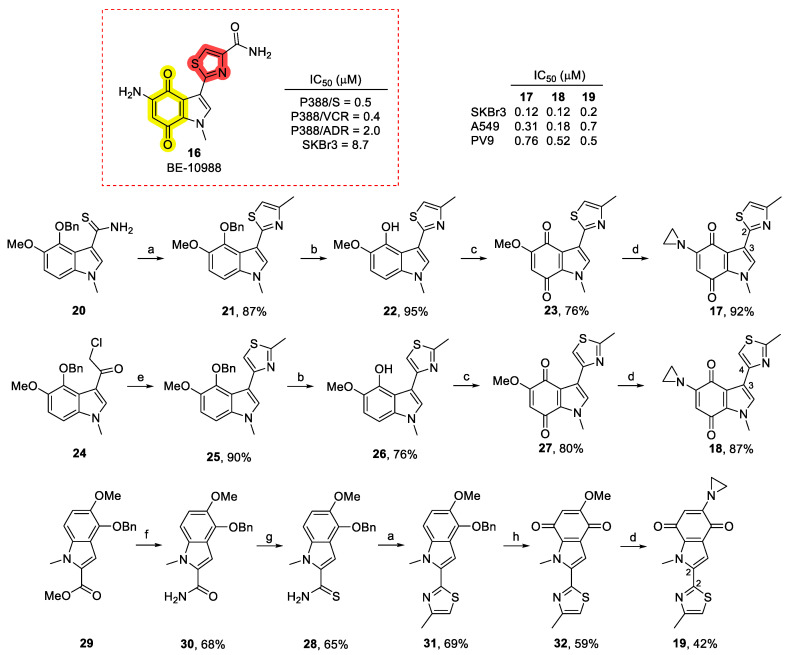
First hybrids of indolequinones and thiazoles tested against carcinogenic strains. (a) BrCH_2_COMe, EtOH, Δ; (b) H_2_, Pd-C, EtOAc; (c) Fremy’s salt, aqueous acetone; (d) aziridine, methanol, rt; (e) 1. MeCSNH_2_, KHCO_3_, 2. (CF_3_CO)_2_O, pyridine; (f) NH_4_OH, heat; (g) Lawesson’s reagent, toluene, Δ; (h) 1. Pd(OH)_2_, H_2_, EtOH, 2. Fremy’s salt.

Only many years later, in the early 1990s, the biological activity of these hybrids began to be studied when the substance BE-10988 (**16**) (Scheme 3), containing the quinone and thiazole moieties linked by a pyrrole ring, was isolated from the fungus *Streptomyces* sp. BA10988. Subsequent studies revealed that this compound presented inhibitory activity of the topoisomerase II enzyme [76].

Quinone **16** was tested against murine lymphoid neoplasm lines (P388/S) and analogous lines resistant to vincristine (P388/VCR) and doxorubicin (P388/ADR), demonstrating a marked inhibition of their growth, with IC_50_ of 0.5, 0.4, and 2.0 μM, respectively [76]. This substance was properly characterized [77] and had its total synthesis carried out [78,79]. These findings motivated the synthesis of indolequinone derivatives varying the substituent linked to the thiazole ring, which had their cytotoxic activity tested against breast (SKBr3) and lung (A549 and PV9) cancer cell lines [80,81]. However, these molecules were not classified as topoisomerase II inhibitors, as they were at least 100-fold less potent than mitoxantrone.

Within this series, the derivatives **17** (IC_50_ = 0.12 μM), **18** (IC_50_ = 0.12 μM), and **19** (IC_50_ = 0.2 μM) bearing aziridyl groups had comparable potency to etoposide and amsacrine against the SKBr3 (human breast) cell line. The derivatives containing the aziridyl group also showed greater cytotoxicity than **16** (IC_50_ = 8.7 μM) against SKBr3. Because the thiazole ring was attached at different positions, different synthetic routes were developed for the production of these compounds (Scheme 2). Quinone **17** features a bond between position 2 of the thiazole and position 3 of the indolequinone. To construct this ring, the strategy adopted was to start with indole **20** (produced from 4-benzyloxy-5-methoxyindole-2-carbaldehyde in 5 steps [78]), which cyclized to **21** using bromoacetone. A reductive deprotection was performed, yielding **22**, which was oxidized with Fremy’s salt to **23**. Finally, the methoxy group was displaced by aziridine, yielding **17**. Quinone **18** has a bond at carbon 4 of the thiazole ring. Synthesis of this molecule began from an indole with a chloroacetyl group **24** (produced from 4-benzyloxy-5-methoxyindole-2-carbaldehyde in 2 steps [78,81]), which, through a modified Hantzsch reaction, yielded the thiazole ring to give compound **25**. The remainder of the route followed the same strategy as the previous methodology. To produce quinone **19**, which has a bond at position 2 of the indolequinone ring, was necessary to obtain the indole-2-thiocarboxamide **28**, starting from ester **29** (produced from 2-benzyloxy-3-methoxybenzaldehyde in 3 steps [82,83]) via amide **30**. In this synthetic route, the thiazole ring was also produced by a Hantzsch reaction, following the same strategy used for **17** and **18**.

**Scheme 4:**
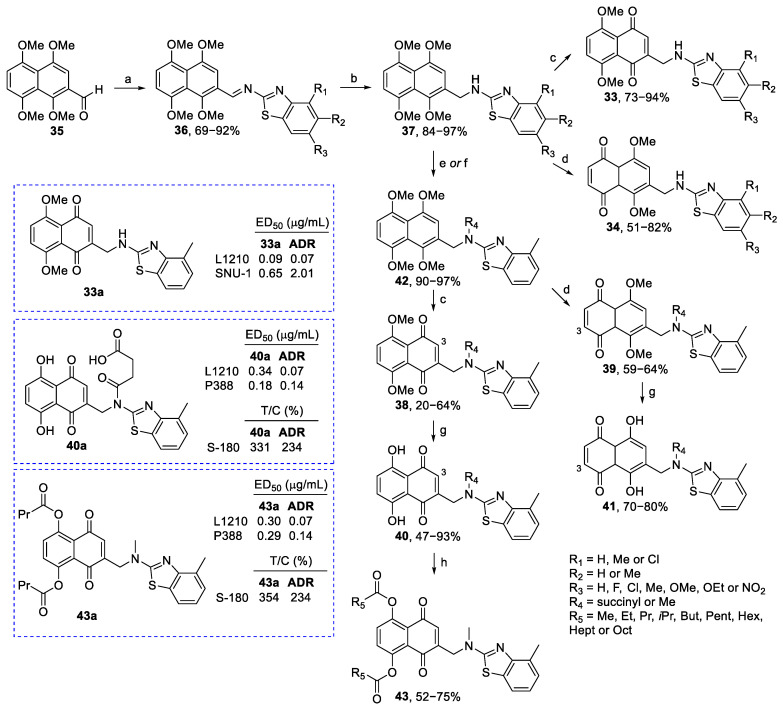
Synthesis of hybrids of naphthoquinone and benzothiazoles **33** and **34**. (a) 2-aminobenzothiazole, TEA, AcOH, Δ; (b) LAH, THF, rt; (c) CAN, MeCN, H_2_O; (d) CrO_3_, acetone, H_2_SO_4_, H_2_O, rt; (e) NaH, THF, MeI, 0−5 °C; (f) succinic anhydride, toluene, Δ; (g) AlCl_3_, DCM, 0–5 °C; (h) acyl chloride, DCM, TEA, 0−5 °C; ADR, adriamycin.

One of the first naphthoquinone–thiazole hybrids with antineoplastic activity, compounds **33** and **34**, were synthesized with an amine bridge linking the two moieties [84]. The synthetic route for producing these molecules (Scheme 4) began with aldehyde **35** (produced from 1,5-naphthalenediol in 4 steps [85,86]), which was condensed with different 2-aminobenzothiazoles to form imines **36**. The imines were reduced with LAH to give amines **37**, which were subsequently oxidized to a mixture of naphthoquinones **33** and **34**. Naphthoquinones **33** were selectively produced using cerium (IV) ammonium nitrate (CAN), and naphthoquinones **34** using chromium (IV) oxide.

The molecules were tested against a lymphocytic leukemia cell line (L1210) and gastric carcinoma (SNU-1). In general, naphthoquinones **34** showed superior activity than their analogues **33**, except for **33a**, which exhibited the highest cytotoxic activity against both cell lines. The antineoplastic activity of **33a** was comparable to that of adriamycin, motivating the synthesis of new naphthoquinone derivatives **38**–**41** with different substituents attached to the amine nitrogen. These derivatives were produced by nucleophilic attack of intermediate amine **37** on methyl iodide or acyl substitution on succinic anhydride [87].

The naphthoquinone moieties of **38** and **39** were produced as previously described, and their respective demethylation in **40** and **41** was performed using AlCl_3_. Derivatives **38**–**41** were tested against L1210 and lymphoid neoplasm (P388). Within this series, **40** and **41**, presenting free hydroxyls, displayed higher activity than their methoxylated analogues **38** and **39**. Interestingly, contrary to what had been previously reported, derivative **40**, in which the thiazole moiety is attached at position 2 of the naphthoquinone, had higher activity than its analogue **41**, attached at position 6. In this context, derivative **40a** showed a rate of growth inhibition (T/C) of 331% in murine S-180 sarcoma cells in the peritoneal cavity, higher than adramycin (234%). Ab initio calculations indicated that substances with greater electrophilicity at carbon 3 tended to show greater cytotoxic activity, as observed for compounds **40** [88]. Continuing the studies with this molecular framework, acyl groups were introduced into the phenolic hydroxyls of **40** using acyl chlorides, producing naphthoquinones **43** [89]. It was found that increasing the carbon chain up to propyl, as in **43a**, enhanced antitumor activity, but continuing to increase the chain resulted in decreased potency.

**Scheme 5:**
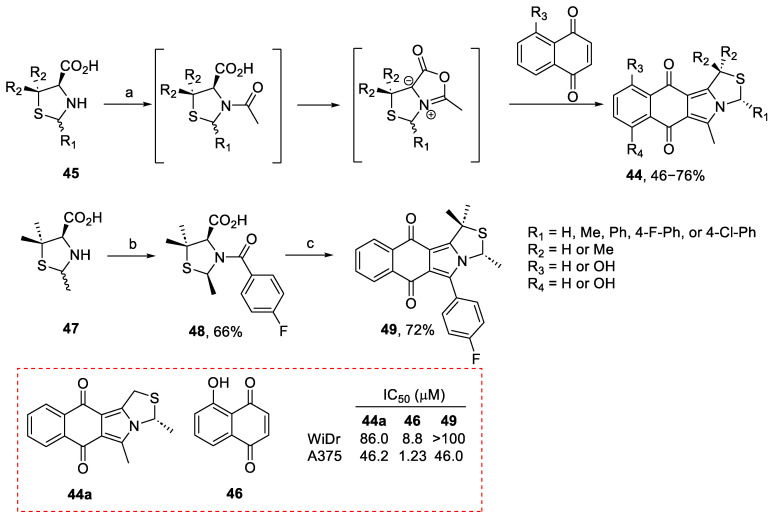
Synthesis of hybrids of naphthoquinones and fused 1*H*,3*H*-pyrrolo[1,2-*c*]thiazoles **44** and **49**. (a) **12** or **46**, Ac_2_O, 120 °C; (b) pyridine, 4-fluoro-benzoyl chloride, 0 °C; (c) **2**, Ac_2_O, 120 °C.

In order to increase the planarity of the molecules to enhance the DNA intercalation process, derivatives **44** were developed, which feature a naphthoquinone fused with a 1*H*,3*H*-pyrrolo[1,2-*c*]thiazole moiety (Scheme 5) [90]. These molecules were produced by heating the corresponding thiazolidine (**45**) in acetic anhydride in the presence of 1,4-naphthoquinone (**12**) or juglone (**46**). The thiazolidines were acylated in situ, followed by a cyclodehydration. Finally, a 1,3-dipolar cycloaddition occurred with **12** or **46**. Acylation of thiazolidine **47** with 4-fluorobenzoyl chloride was also performed, yielding **48** as a pure diastereoisomer. Compound **48** was then used to produce the naphthoquinone derivative **49**. All derivatives **44** were isolated as pure *R* enantiomers. These molecules were tested against colorectal adenocarcinoma (WiDr) and melanoma (A375) cell lines but, except for **44a**, were inactive against WiDr. Derivatives **44** with hydroxyls in the aromatic ring were inactive against both strains, which contrasts with their parent naphthoquinone, **46**, that is active against WiDr (IC_50_ = 8.8 μM) and A375 (IC_50_ = 1.23 μM). The results demonstrated that the fusion of the quinone ring with thiazolidine resulted in decreased antineoplastic activity for these cell lines.

**Scheme 6:**
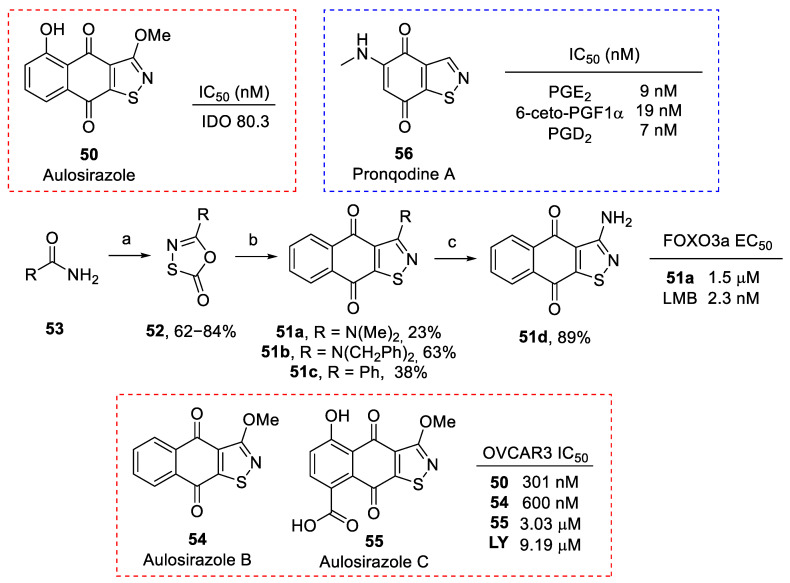
Synthesis of hybrids of isothiazolonaphthoquinones **51**. (a) chlorocarbonylsulphenyl chloride, *N*,*N*-dimethylurea or *N*,*N*-dibenzylurea or benzamide, MeCN or toluene, rt or reflux; (b) 1,4-naphthoquinone (**12**), xylene, reflux; (c) CAN, MeCN/H_2_O (9:1), rt. LMB, leptomycin B. LY, LY294002.

Although studies on the antineoplastic activity of naphthoquinone-isothiazole hybrids are less common, the natural compound aulosirazole (**50**) (Scheme 6), after isolation from the algae *Aulosira fertilissima*, was identified as a promising antineoplastic molecule by exhibiting selective activity in solid tumors in the Corbett assay [91]. The antineoplastic potential of the molecular scaffold of **50** subsequently motivated investigations of this compound and its analogues as a potential inhibitor of the enzyme indoleamine-2,3-dioxygenase [92].

Derivatives **51**, which contain an isothiazole ring fused to a quinonoid core, were identified within a library of molecules designed as potential histone deacetylase (HDAC) inhibitors, an important class of targets in antineoplastic drug development [93]. Molecules **51** are structurally analogous to **50** and were able to decrease the viability of LN18 and T98 glioma cells at a concentration of 20 μM. FOXO protein activation is an interesting target for the development of bioactive molecules against tumors due to its tumor suppressive activity [94]. Hundreds of compounds were tested for their ability to induce nuclear translocation of the GFP-FOXO3a reporter protein in the U2OS (osteosarcoma) cell line at a concentration of 10 μM [95]. Among them, **51a** induced nuclear FOXO translocation with an EC_50_ of 1.5 μM. Interestingly, compound **51d**, which lacks the two methyl groups attached to the nitrogen atom, did not induce nuclear accumulation of FOXO fluorescent reporter protein at 10 μM, suggesting that the dimethylamino group may be crucial for interaction with the biological target.

Derivatives **51** were produced from 1,3,4-oxathiazol-2-ones **52** (Scheme 6) [93], which were prepared from the reaction between ureas or benzamide (**53**) and chlorocarbonylsulfynyl chloride. Thermal decarboxylation of **52** generated nitrile sulfides that reacted with 1,4-naphthoquinone (**12**) in a 1,3-dipolar cycloaddition. Naphthoquinone **51b** also underwent debenzylation with CAN, yielding **51d**.

Aulosirazole (**50**) was also isolated from *Nostoc* sp. UIC 10771 [96]. Along with **50**, two other related natural compounds, aulosirazole B (**54**) and aulosirazole C (**55**) (Scheme 6), were also identified in the cyanobacteria. Aulosirazole (**50**) and both analogues were tested against the ovarian cancer cell line OVCAR3. Aulosirazole (**50**) was the most potent molecule (IC_50_ = 301 nM), followed by **54** (IC_50_ = 600 nM) and **55** (IC_50_ = 3.03 μM). Owing to its high potency, the ability of **50** to induce nuclear accumulation of FOXO3a was further investigated. This molecule induced a significant nuclear accumulation accompanied by a reduction in its cytosolic concentration of FOXO3a when compared to positive and vehicle controls.

Another natural compound with quinonoid and isothiazole moieties is pronqodine A (**56**) (Scheme 6), isolated from *Streptomyces* sp. MK832-95F2 [97]. This molecule inhibited bradykinin-induced prostaglandin (PG) release in a concentration-dependent manner in human synovial sarcoma (SW982) cells. Inhibition occurred with IC_50_ values of 9 nM for PGE2 production, 19 nM for 6-keto-prostaglandin F1α, and 7 nM for PGD2. Data from mechanistic studies suggest that **56** acts as a bioreductant, inhibiting prostaglandin release in activated NQO1-expressing cells.

**Figure 3:**
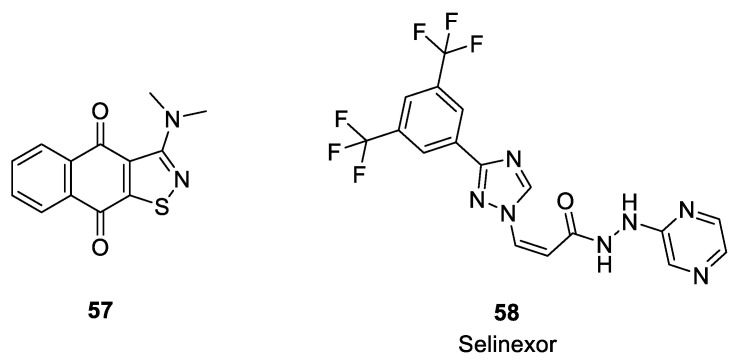
Chemical structures of isothiazolonaphthoquinone-based compound (**57**) and Selinexor (**58**).

A study examined the synergistic anticancer potential of the isothiazolonaphthoquinone-based compound **57**, an activator of FOXO nuclear–cytoplasmic shuttling, in combination with selinexor (**58**), an exportin-1 (XPO1) inhibitor, against breast cancer (Figure 3) [98]. Compound **57** promoted the nuclear accumulation of the transcription factor FOXO1, a tumor suppressor whose activity is tightly linked to its subcellular localization. In vitro assays on MCF-7 and MDA-MB-175 breast cancer cell lines revealed that **57** alone significantly enhanced FOXO1 nuclear localization, leading to inhibition of cell migration, induction of apoptosis, and downregulation of oncogenic proteins c-Myc and cyclin D1. When combined with selinexor (**58**), these effects were markedly potentiated, exhibiting strong synergism (combination index < 0.9). Mechanistically, the dual treatment promoted FOXO1 competition with TCF for β-catenin binding, resulting in suppression of the Wnt/β-catenin signaling pathway, a key regulator of tumor proliferation and metastasis. In vivo, co-administration of both compounds in MCF-7-derived xenografts led to superior tumor growth inhibition (>40% TGI) without systemic toxicity, outperforming either monotherapy.

**Scheme 7:**
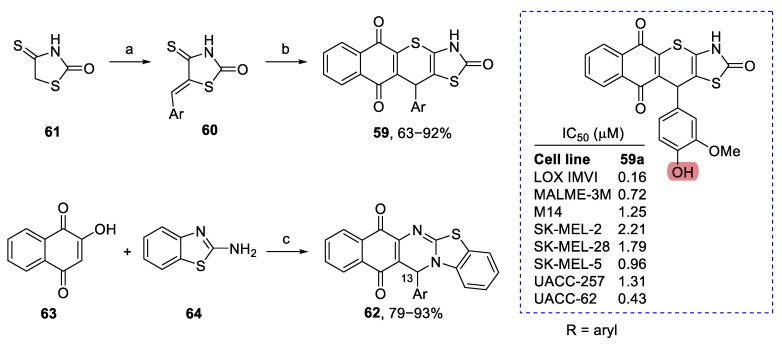
Synthesis of hybrids **59** and **62**. (a) ArCHO, AcONa, AcOH, 100 °C; (b) 1,4-naphthoquinone (**12**), AcOH, hydroquinone (cat.), reflux; (c) aromatic aldehyde, amberlyst−15, 100 °C.

Continuing with naphthoquinones with fused heterocycles, the fusion of the quinonoid ring with thiopyrano[2,3-*d*]thiazol-2-one led to the synthesis of derivatives **59** (Scheme 7) [99]. Thiazolidines **60** were prepared by treatment of 4-thioxo-2-thiazolidinone **61** with the corresponding aromatic aldehyde. Compounds **59** were synthesized by hetero-Diels–Alder reaction between **60** and 1,4-naphthoquinone (**12**). The synthesized hybrids were tested on several cell lines, with melanoma cell lines being the most sensitive, especially **59a**. The superior activity of **59a** was attributed to the presence of the hydroxyl in the *para* position, since its methylation decreased the antineoplastic activity.

Another series of compounds featuring a six-membered ring fused to the quinonoid ring, **62**, was synthesized via a multicomponent reaction between lawsone (**63**), aromatic aldehydes, and 2-amino-benthiazole (**64**) (Scheme 7) [100]. The compounds were tested against hepatocellular carcinoma (HepG2) and cervical cancer (HeLa) cell lines. Although none of the derivatives had hydroxyl groups in the aromatic ring, it was found that the presence of electron-donating groups increased the cytotoxic activity. Additionally, the results suggested that the rotation of the aromatic ring at position 13 may be important for the activity, since 2,5-disubstituted derivatives had lower cytotoxicity.

**Scheme 8:**
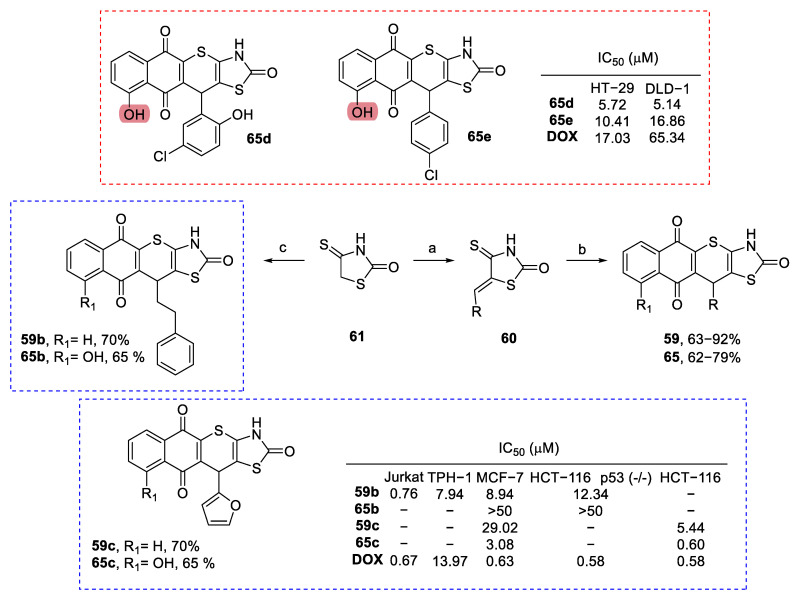
Synthesis of hybrids **59** and **65**. (a) aldehyde or ketone, EDDA, EtOH, reflux, 10 min; (b) 1,4-naphthoquinone (**12**) or juglone (**46**), AcOH, hydroquinone (cat.), reflux; (c) phenylpropionaldehyde, 1,4-naphthoquinone (**12**) or juglone (**46**), EDDA, MeCN, reflux. DOX, doxorubicin.

In the context of 1,4-naphthoquinones with thiopyrano[2,3-*d*]thiazol-2-one, new derivatives of type **59** were reported by Lozynsky and co-workers (Scheme 8), with particular emphasis on compound **59b**, which was obtained through a multicomponent reaction involving **61**, phenylpropionaldehyde, and 1,4-naphthoquinone (**12**) [101]. This derivative exhibited the most pronounced biological effects within the series, especially against leukemia cell lines, such as Jurkat (IC_50_ = 0.76 µM), comparable to the reference drug doxorubicin (IC_50_ = 0.67 µM), and TPH-1 (IC_50_ = 7.94 µM), being approximately twice as potent as doxorubicin (IC_50_ = 13.97 µM). Moreover, compound **59b** induced pro-apoptotic cytomorphological alterations, mitotic catastrophe in treated KB3-1 cells, necrotic cell death, and demonstrated direct interaction with DNA.

Subsequently, the same research group conducted further studies employing juglone (**46**) as a synthetic platform to obtain a new series of analogs related to **59**, featuring a hydroxyl group on the aromatic ring derived from the naphthoquinone moiety (Scheme 8) [102]. From a biological perspective, compound **65b** did not exhibit cytotoxic activity comparable to its counterpart **59b**. Although not tested across all the same cancer cell lines, the presence of the hydroxyl group proved detrimental to activity, as observed for MCF-7 (**59b**: IC_50_ = 8.94 µM; **65b**: IC_50_ > 50 µM) and HCT-116 p53 (−/−) (**59b**: IC_50_ = 12.34 µM; **65b**: IC_50_ > 50 µM). Interestingly, this trend was reversed for the pair of compounds **59c** and **65c**, both bearing R = furan-2-yl, where **65c** exhibited superior cytotoxicity across all tested cell lines compared to its non-hydroxylated analog **59c**. Notably, **65c** emerged as the most potent derivative of the new series, particularly against the HCT-116 cell line, with an IC_50_ of 0.60 µM, comparable to the reference drug doxorubicin (IC_50_ = 0.58 µM) and approximately 10-fold more active than its corresponding non-hydroxylated analog **47c** (IC_50_ = 5.44 µM).

Continuing the investigation of juglone-bearing thiopyrano[2,3-*d*]thiazole derivatives, Kozak and co-workers report the synthesis and biological evaluation of two compounds of type **65** obtained through a regioselective hetero-Diels–Alder reaction between juglone (**46**) and substituted 4-thioxothiazolidin-2-ones, similar to that illustrated in Scheme 8 [103]. Both compounds exhibited favorable in silico ADMET properties and strong binding affinities in molecular docking studies, particularly toward CDK2, JAK2, and MAPK8, which are key targets involved in cancer proliferation and apoptosis. In vitro assays demonstrated potent and selective cytotoxicity of **65d** and **65e** against colorectal adenocarcinoma cell lines HT-29 and DLD-1, with IC_50_ values ranging from 1.9 to 10.4 µM, while exhibiting low toxicity toward pseudonormal and normal human cells. Mechanistic studies revealed that both compounds induced significant ROS generation, S-phase and G2/M cell cycle arrest, and activation of both intrinsic and extrinsic apoptotic pathways via caspase-3/7, -8, -9, and -10 activation.

**Scheme 9:**
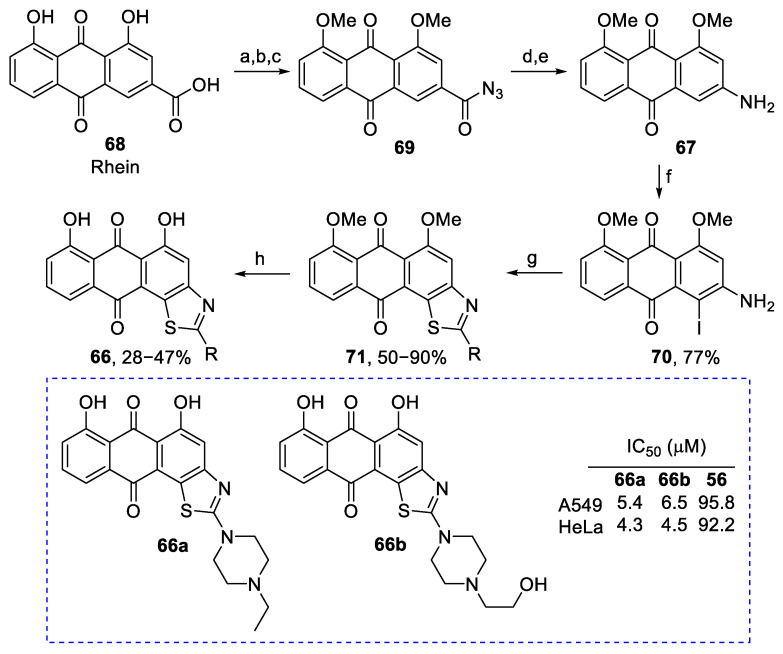
Synthesis of thiazole-fused anthraquinones **66**. (a) NaH, MeI, DMF, ice bath, overnight; (b) NaOH in H_2_O/EtOH, 50 °C, 1 h; (c) Et_3_N, DPPA, DMF, rt, 1 h; (d) dioxane, reflux, 30 min; (e) NaOH in H2O, reflux, 4 h; (f) I_2_, Ag_2_SO_4_, ethylene glycol, rt, 3 h; (g) amine, CS_2_, K_2_CO_3_, DMF, 115 °C, 6 h; (h) EtSH, AlCl_3_, DCM, rt, 10 h or BBr_3_, DCM, rt, 10 h.

Within the quinone family, anthraquinones stand out for their antineoplastic capacity [104], as evidenced by the anthracycline class of chemotherapeutics widely used for the treatment of various types of cancer. The recognized antineoplastic activity of anthraquinones, combined with evidence that fusion with heterocyclic moieties, including imidazoles, generates bioactive compounds of interest, inspired the synthesis of anthraquinone derivatives fused to thiazole **66** [105]. Scheme 9 describes the synthesis process of these derivatives. The amino intermediate **67** was obtained by a Curtius rearrangement from rhein (**68**) via the acyl azide intermediate **69**. Selective iodination of **67** using iodine and silver sulfate afforded intermediate **70**. The thiazole moiety of **71** was constructed by the reaction of **70** with different amines in a medium containing carbon disulfide. Finally, demethylation of **71** to give **66** occurred using EtSH/AlCl_3_ or BBr_3_ at room temperature. Most of the compounds tested against lung cancer (A549) and cervical cancer (HeLa) cell lines exhibited higher cytotoxicity than the starting material rhein (**56**), with **66a** and **66b** being the most potent. Within this series, derivatives containing more than one heteroatom in the side chain of the thiazole ring generally showed better activities, possibly due to improved aqueous solubility.

**Scheme 10:**
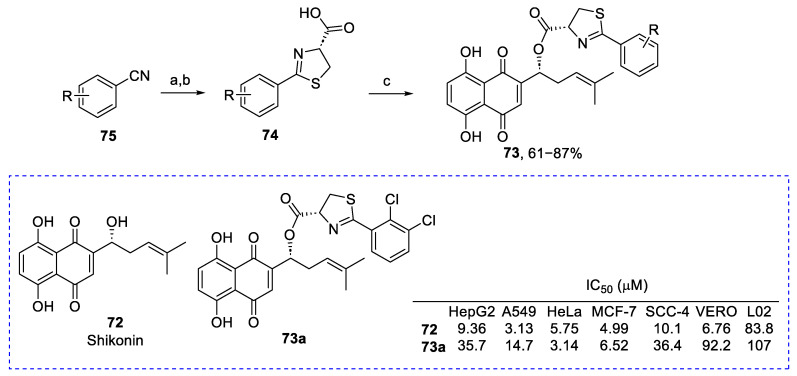
Synthesis of thiazole derivatives of shikonin **73**. (a) L-cysteine, NaHCO_3_, MeOH/H_2_O, NaOH, rt; (b) L-cysteine, NaHCO_3_, EtOH, 100 °C; (c) Shikonin (**72**), DMAP, DCC, DCM, rt, 12 h.

Shikonin (**72**) is a naphthoquinone with excellent antineoplastic activity [106,107,108], and evidence suggests that esterification of its aliphatic hydroxyl enhances this activity [109,110]. Considering these factors, hybrids **73** of shikonin (**72**) and thiazoles **74** connected by an ester bridge were synthesized [111]. Initially, the thiazole moiety of **74** was constructed by a cyclization between different aryl nitriles (**75**) and L-cysteine (Scheme 10). Then, the esterification between the acidic group and the aliphatic hydroxyl was performed using 4-dimethyaminopyridine (DMAP) and *N*,*N*’-dicyclohexylcarbodiimide (DCC). The presence of the dihydrothiazole group in **73**, in general, translated into a lower antineoplastic activity when compared to **72**, but conferred greater selectivity toward tumor cell lines. Among the **60** derivatives prepared, compound **73a** showed greater cytotoxicity against the HeLa cell line (IC_50_ = 3.14 μM) in addition to a lower activity against the non-tumor cell line VERO (IC_50_ = 92.2 μM) in comparison to **72** (IC_50_ HeLa = 5.75 μM; IC_50_ VERO = 6.76 μM). Docking and confocal microscopy experiments were performed, which indicated that **73a** could bind to tubulin at the paclitaxel binding site, leading to tubulin polymerization and mitotic rupture.

**Scheme 11:**
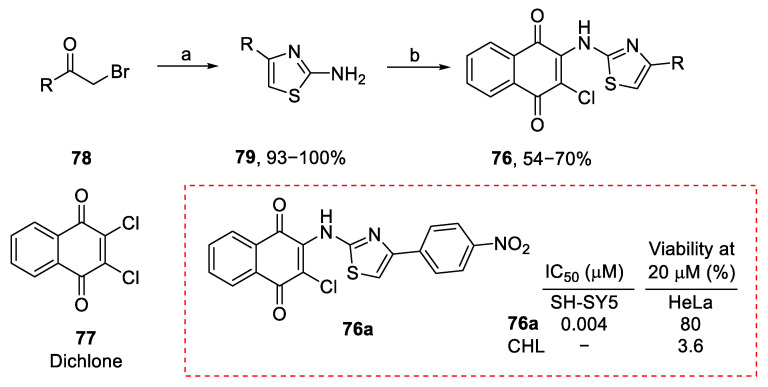
Synthesis of thiazole derivatives of dichlone **76**. (a) thiourea, EtOH, 70 °C, 1 h; (b) **77** (dichlone), K_2_CO_3_, DMF, rt, 5 h. CHL, chloroquine.

The biological activities of naphthoquinone and thiazole hybrids have already been demonstrated throughout this review. In particular, derivatives **59** [99] inspired the synthesis of thiazole derivatives **76** of dichlone (**77**) [112]. The synthesis began with a Hantzsch condensation between α-haloketones **78** and thiourea, producing 2-aminothiazoles **79**. These intermediates then underwent nucleophilic vinylic substitution in **77**, providing derivatives **76** (Scheme 11). These molecules were tested against HeLa and SH-SY5 tumor cell lines. Against the SH-SY5 cell line, it was observed that electron-withdrawing groups were associated with greater antineoplastic activity, as was the case for derivative **76a**, R = p-NO_2_Ph (IC_50_ = 0.004 μM), when compared with **76b**, R = Ph (IC_50_ = 1.8 μM). However, these derivatives did not demonstrate considerable activity against HeLa cells.

**Scheme 12:**
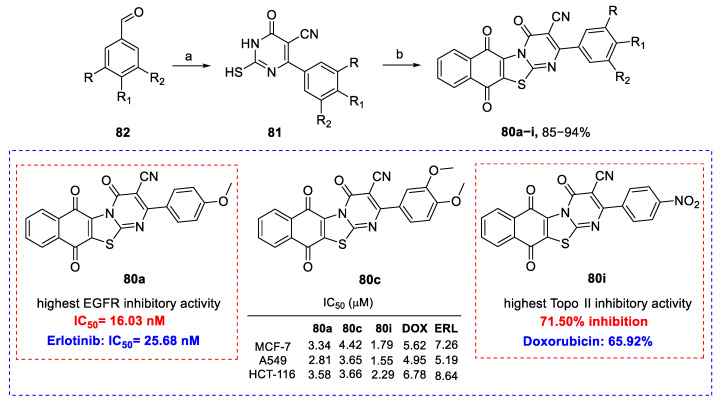
Synthesis of naphtho[2′,3′:4,5]thiazolo[3,2-*a*]pyrimidine hybrids **80**. (a) thiourea, ethyl cyanoacetate, K_2_CO_3_, EtOH, reflux, 2 h; (b) **77** (dichlone), DMF, rt, 12 h. DOX, doxorubicin and ERL, erlotinib.

A series of naphtho[2′,3′:4,5]thiazolo[3,2-a]pyrimidine hybrids **80** was successfully synthesized through molecular hybridization of thiazolopyrimidine and naphthoquinone scaffolds. The synthetic strategy started with the preparation of 6-aryl-2-mercapto-1,6-dihydropyrimidine-5-carbonitriles (**81**) via modified Biginelli condensation of ethyl cyanoacetate, diverse aromatic aldehydes (**82**), and thiourea, employing potassium carbonate as the base [113]. The target derivatives (**80**) were subsequently obtained via the reaction of thiazolopyrimidines (**81**) with dichlone (**77**), as shown in Scheme 12.

Biological evaluation revealed that several derivatives, particularly **80a**, **80c**, and **80i**, exhibited superior cytotoxicity against MCF-7, A549, and HCT-116 cancer cell lines when compared with doxorubicin and erlotinib, used as reference drugs. Hybrid **80i** demonstrated the most potent cytotoxic activity, with IC_50_ values ranging from 1.55 to 2.29 µM, making it approximately three times more active than doxorubicin and up to four times more active than erlotinib, as shown in Scheme 12. Mechanistic studies demonstrated that these hybrids act as dual inhibitors of topoisomerase IIα and EGFR. They function as catalytic inhibitors of topo IIα while effectively suppressing EGFR kinase activity, with compound **80a** showing nanomolar potency (IC_50_ = 16.03 nM). Furthermore, these derivatives induced apoptosis through upregulation of p53 and pro-apoptotic protein Bax, downregulation of anti-apoptotic protein Bcl-2, and activation of caspases-7 and -9, most prominently for compound **80i**. Molecular docking supported strong binding interactions with the ATPase domain of topo IIα and the active site of EGFR, in line with the observed multitarget activity. Collectively, these findings identify compounds **80a**, **80c**, and **80i** as promising dual-acting anticancer prototypes with potential for further development as clinically relevant multitarget agents.

**Scheme 13:**
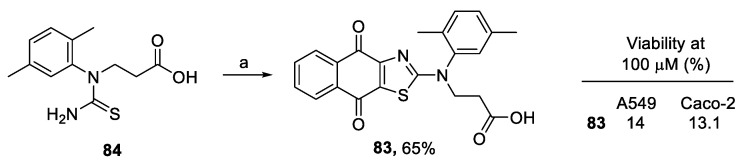
Synthesis of thiazole-fused 1,4-naphthoquinone **83**. (a) **77** (dichlone), sodium acetate, glacial acetic acid, rt, 24 h, then 70–80 °C, 12 h.

A diverse library of thiazole derivatives incorporating the *N*-2,5-dimethylphenylthioureido acid scaffold was synthesized, leading to the identification of compound **83**, a naphthoquinone-fused thiazole with promising anticancer potential [114]. Structurally, the fusion of the thiazole core with a naphthoquinone moiety conferred interesting biological activity, underscoring the importance of extended conjugation and redox-active substituents in enhancing cytotoxic properties. Compound **83** exhibited broad-spectrum anticancer activity, significantly reducing the viability of both pulmonary adenocarcinoma (A549) and colorectal adenocarcinoma (Caco-2) cell lines to 14% and 13.1%, respectively, after 24 h of exposure, compared to untreated control (Scheme 13).

**Scheme 14:**
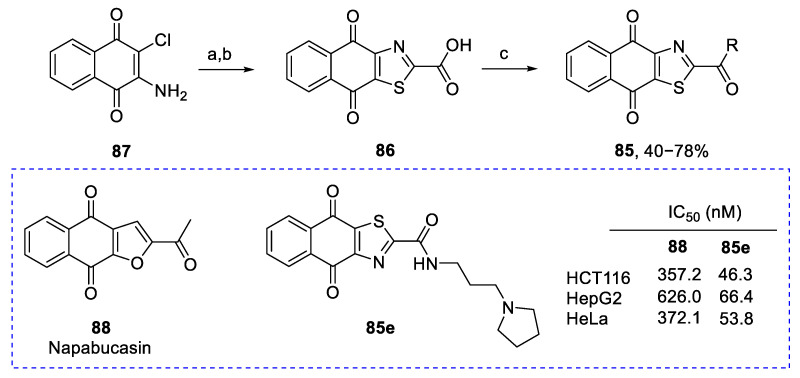
Synthesis of naphthoquinothiazole derivatives **85**. (a) Na_2_S·9H_2_O, EtOH/H_2_O, 90 °C; (b) Glyoxylic acid, 0 °C; (c) NH_2_R, HATU, TEA, DMF.

A new series of naphthoquinothiazole derivatives was designed and synthesized through scaffold modification of napabucasin (**88**), aiming to enhance STAT3 inhibition and redox modulation [115]. Among the compounds, **85e** demonstrated the most potent anticancer activity, with IC_50_ values of 46.3 nM, 66.4 nM, and 53.8 nM against HCT116, HepG2, and HeLa cells, respectively, while maintaining low cytotoxicity toward normal fibroblasts, resulting in a high selectivity index. Mechanistic studies revealed that **85e** induces S-phase arrest, apoptosis, and strong intracellular ROS production, accompanied by suppression of AKT phosphorylation. Furthermore, **85e** effectively inhibited STAT3 phosphorylation at Tyr705, with direct binding confirmed by SPR and CETSA assays, and docking simulations showed stable interactions with the STAT3 SH2 domain. Importantly, in vivo experiments using HCT116 xenografts confirmed that **85e** significantly suppressed tumor growth without observable toxicity (Scheme 14).

A high-throughput screening of 5700 compounds from the National Cancer Institute (NCI) chemical library using *Schizosaccharomyces pombe* deletion mutants enabled the identification of several potential anticancer agents. Among these, compound **89** emerged as particularly promising because it exhibited lower IC_50_ values against *S. pombe* and demonstrated higher cytotoxicity toward yeast cells than toward mammalian cells, suggesting that these compound may selectively target yeast-specific pathways and hold potential for further development as an anticancer agents [116].

**Scheme 15:**
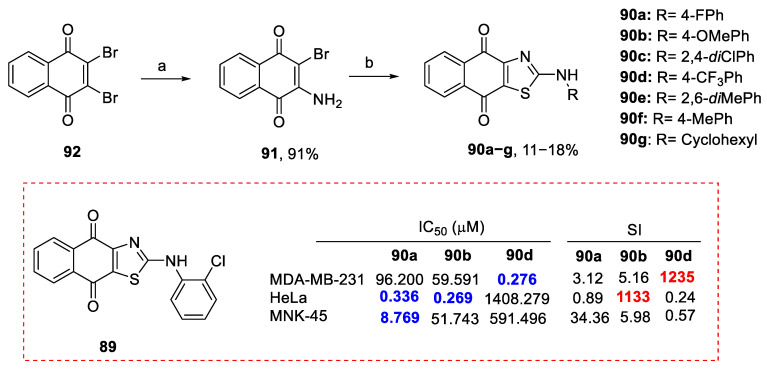
Synthesis of 2-aminonaphtho[2,3-*d*][1,3]thiazole-4,9-dione derivatives **90**. (a) NH_3_, H_2_O, dioxane, 2.5 h, rt; (b) isothiocyanate derivatives, CuO nanoparticles, K_2_CO_3_, H_2_O, 24 h, reflux.

Inspired by these results, Yanik and coworkers synthesized a series of 2-aminonaphtho[2,3-*d*][1,3]thiazole-4,9-dione derivatives (**90**) via the condensation of 2-amino-3-bromo-1,4-naphthoquinone (**91**) with various isothiocyanates under CuO nanoparticle-catalyzed conditions, forming of thiourea intermediates that cyclized to yield the desired heterocycles (Scheme 15) [117].

The compounds were evaluated for their cytotoxic potential against MDA-MB-231 (breast), HeLa (cervical), and MKN-45 (gastric) cancer cell lines using MTT assays. Among them, compounds **90b** and **90d** displayed the most potent antiproliferative activity, with IC_50_ values of 0.269 μM (HeLa) and 0.276 μM (MDA-MB-231), respectively, and high selectivity index values. Compound **90a** also showed strong inhibition in HeLa (IC_50_ = 0.336 μM) and MKN-45 (IC_50_ = 8.769 μM) cells. Molecular docking studies suggested favorable binding of the ligands to the human DNA topoisomerase IIβ (hDNA TopoIIβ) active site, stabilized by hydrogen bonding and cation–π interactions, supporting a potential mechanism of TopoIIβ inhibition.

**Scheme 16:**
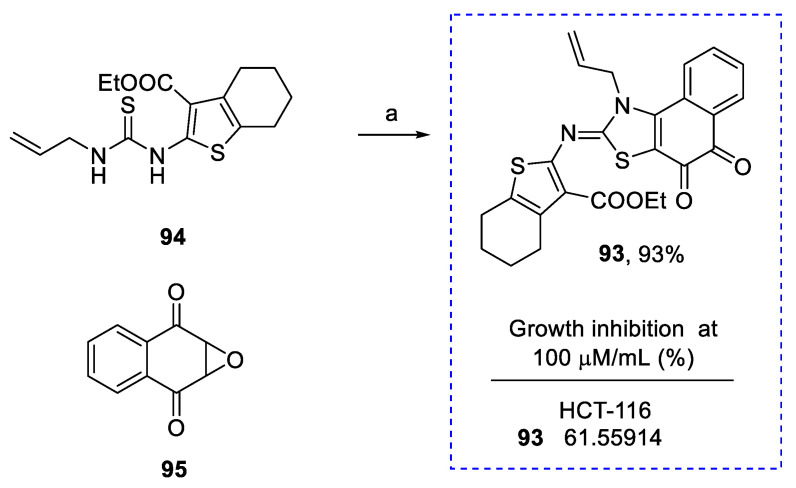
Synthesis of ethyl (*Z*)-2-((1-allyl-4,5-dioxo-4,5-dihydronaph tho[1,2-*d*]thiazol-2(1*H*)-ylidene)amino)-4,5,6,7-tetrahydrobenzo[*b*]thiophene-3-carboxylate **93**. (a) **95**, EtOH, reflux, 5 h.

A new series of thiophene-based heterocycles incorporating thioureido substituents was synthesized, among which only ethyl (*Z*)-2-((1-allyl-4,5-dioxo-4,5-dihydronaphtho[1,2-*d*]thiazol-2(1*H*)-ylidene)amino)-4,5,6,7-tetrahydrobenzo[*b*]thiophene-3-carboxylate (**93**) has the naphthoquinone core. Compound **93** was obtained by reaction of intermediate **94** and 2,3-dihydro-2,3-epoxy-1,4-naphthoquinone (**95**) [118]. In vitro assays revealed that these derivatives exhibited strong growth inhibition toward HCT-116 cells (61.55914% for **93**). However, the IC_50_ was not reported for compound **93** (Scheme 16).

**Scheme 17:**
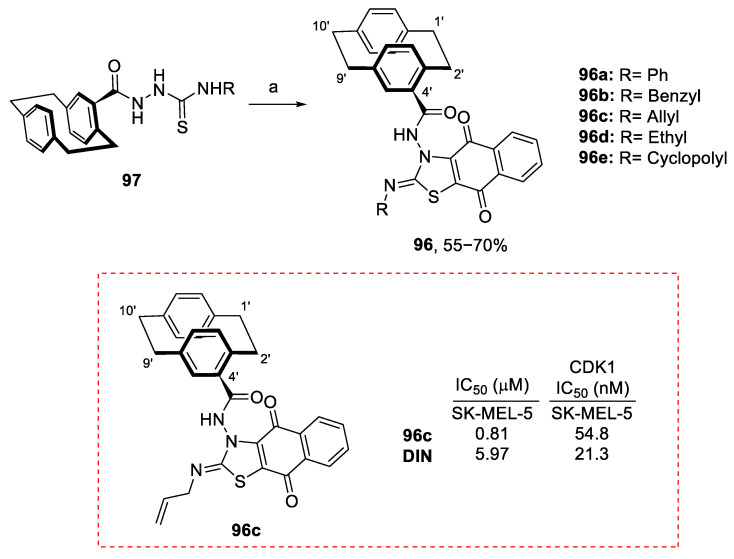
Synthesis of paracyclophanyl–thiazole naphthoquinone hybrids **96**. (a) **64**, PhP_3_, Et_3_N, CH_3_CN, reflux, 10–14 h. DIN, dinaciclib.

A series of paracyclophanyl–thiazole hybrids incorporating a naphthoquinone scaffold was designed and synthesized as potential cyclin-dependent kinase 1 (CDK1) inhibitors, exhibiting pronounced antiproliferative activity against melanoma cells. The target compounds (**96**) were synthesized via the Eschenmoser coupling reaction of *N*-substituted thioureas (**97**) with dichlone (**77**), affording the desired conjugates in satisfactory yields (Scheme 17) [119].

Comprehensive biological screening conducted by the NCI demonstrated that these derivatives exhibited strong cytotoxic profiles across a broad spectrum of cancer cell lines, with **96c**, **96d**, and **96e** inducing complete growth inhibition in several panels, notably melanoma, colon, and CNS cancers. Among them, compound **96c** emerged as the most potent, displaying an IC_50_ value of 0.81 μM against the SK-MEL-5 melanoma cell line, markedly superior to the reference CDK inhibitor dinaciclib (IC_50_ = 5.97 μM). Mechanistic investigations revealed that **96c** selectively inhibited CDK1 (IC_50_ = 54.8 nM), promoted cell-cycle arrest at the G_2_/M phase, and induced apoptosis via caspase-3 overexpression and phospho-Tyr15 downregulation. Molecular docking studies further supported these findings, highlighting strong hydrogen-bonding interactions with GLN137 and ILE15, together with enhanced π–π stacking interactions mediated by the paracyclophane moiety.

**Scheme 18:**
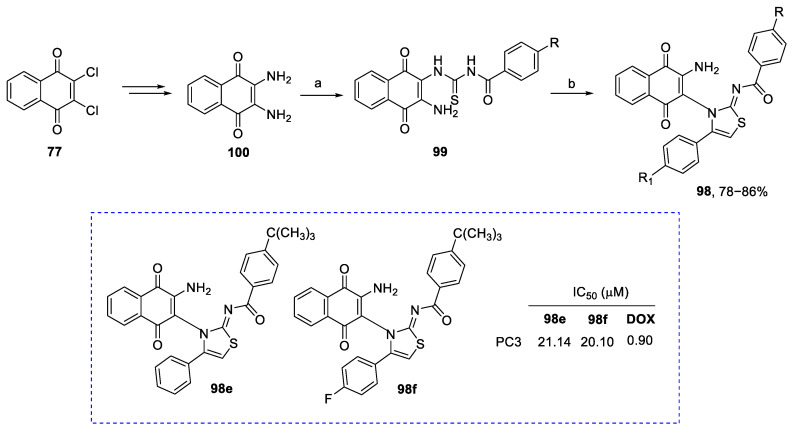
Synthesis of 1,4-naphthoquinone–thiazole hybrids **98**. (a) isothiocyanates, acetone, reflux, 18 h; (b) α-bromoketones, acetone, reflux, 12 h. DOX, doxorubicin.

A series of 1,4-naphthoquinone–thiazole hybrids (**98**) was synthesized through the cyclization of 1,4-naphthoquinone thioureas (**99**) with α-bromoketones in good yields (Scheme 18) [120]. The hybrids were evaluated for antiproliferative activity against PC3 human prostate cancer cells, with compounds **98f** (IC_50_ = 20.10 µM) and **98e** (IC_50_ = 21.14 µM) showing the highest cytotoxic effects. Hoechst staining confirmed that **98f** induces apoptosis in a dose-dependent manner. Inverse molecular docking and molecular dynamics simulations suggested that the anticancer mechanism of **98f** is likely mediated through p38 MAP-kinase inhibition, with stable binding confirmed over 300 ns. Additionally, solubility studies revealed that derivatives bearing a fluorine atom at R_1_ and a *tert*-butyl substituent at R display improved solubility and biological performance.

## 3. Conclusions

In summary, naphthoquinone–thiazole hybrids clearly demonstrate their relevance as privileged scaffolds in anticancer drug discovery. The combination of the redox-active naphthoquinone core with the pharmacophoric versatility of the thiazole ring has generated a structurally diverse range of compounds with notable cytotoxic profiles. From a medicinal chemistry perspective, molecular hybridization has proven to be an effective strategy for designing multitarget agents capable of overcoming common limitations of conventional chemotherapeutics, such as resistance and systemic toxicity. Continued exploration of structural modifications, mechanistic pathways and biological validations will be essential to advance these hybrid frameworks toward clinically relevant anticancer agents.

## Data Availability

No new data were created or analyzed in this study. Data sharing is not applicable to this article.
